# Raman Spectroscopic Imaging of Human Bladder Resectates towards Intraoperative Cancer Assessment

**DOI:** 10.3390/cancers15072162

**Published:** 2023-04-05

**Authors:** Christoph Krafft, Jürgen Popp, Peter Bronsert, Arkadiusz Miernik

**Affiliations:** 1 Leibniz Institute of Photonic Technology, Member of Leibniz Health Technologies and Member of the Leibniz Centre for Photonics in Infection Research, 07745 Jena, Germany; 2 Institute of Physical Chemistry and Abbe Center of Photonics, Friedrich Schiller University Jena, Member of the Leibniz Centre for Photonics in Infection Research, 07743 Jena, Germany; 3 Medical Center, Faculty of Medicine, Institute of Surgical Pathology, University of Freiburg, 79106 Freiburg, Germany; 4 Medical Center, Faculty of Medicine, Department of Urology, University of Freiburg, 79106 Freiburg, Germany

**Keywords:** Raman spectroscopy, bladder cancer, hyperspectral unmixing, microplastic, pigment

## Abstract

**Simple Summary:**

Bladder cancer is the sixth most incident neoplasm, and the average five-year survival rate drops to 5% for patients with the metastatic disease. Raman spectroscopy probes molecular vibrations by inelastic scattering of laser light. In a retrospective study, control and cancer specimens were prepared from ten human bladder resectates and label-free assessed by Raman microspectroscopic imaging. A data analysis workflow without preprocessing identified the locations and signatures of normal bladder, bladder cancer, necrosis, epithelium, and lipid inclusions. Furthermore, microplastic particles, pigments or carotenoids were detected in 13 out of 20 specimens inside tissue and near tissue margins and their identity was confirmed by spectral library surveys. Hypotheses about the origin of these foreign materials are discussed. Our approach with minimal user interference offers prospects for future clinical translation such as intraoperative tumor detection and label-free material identification in complex matrices.

**Abstract:**

Raman spectroscopy offers label-free assessment of bladder tissue for in vivo and ex vivo intraoperative applications. In a retrospective study, control and cancer specimens were prepared from ten human bladder resectates. Raman microspectroscopic images were collected from whole tissue samples in a closed chamber at 785 nm laser excitation using a 20× objective lens and 250 µm step size. Without further preprocessing, Raman images were decomposed by the hyperspectral unmixing algorithm vertex component analysis into endmember spectra and their abundancies. Hierarchical cluster analysis distinguished endmember Raman spectra that were assigned to normal bladder, bladder cancer, necrosis, epithelium and lipid inclusions. Interestingly, Raman spectra of microplastic particles, pigments or carotenoids were detected in 13 out of 20 specimens inside tissue and near tissue margins and their identity was confirmed by spectral library surveys. Hypotheses about the origin of these foreign materials are discussed. In conclusion, our Raman workflow and data processing protocol with minimal user interference offers advantages for future clinical translation such as intraoperative tumor detection and label-free material identification in complex matrices.

## 1. Introduction

Bladder cancer is the sixth most incident neoplasm in the United States, and the disease is four times more common in men than women. Bladder cancer ranges from unaggressive and usually noninvasive tumors that recur and commit patients to long-term invasive surveillance to aggressive and invasive tumors. The average five-year survival in the United States is 77%, and drops to 5% for those with the metastatic disease [1]. Bladder cancers can present with gross or microscopic hematuria, which is evaluated with cystoscopy and upper tract imaging. Non-muscle-invasive tumors are treated with endoscopic resection and adjuvant intravesical therapy. Patients with high-risk non-muscle invasive tumors that do not respond to adjuvant therapy with the standard-of-care immunotherapy constitute a challenging population to manage, and many alternative therapies are being studied [2]. For patients with muscle-invasive disease, more aggressive therapy with radical cystectomy and urinary diversion or trimodal therapy with maximal endoscopic resection, radiosensitizing chemotherapy, and radiation is warranted to curb the risk of metastasis and disease-specific mortality [3]. Photodynamic therapy (PDT) is one of the few treatment options for patients with recurrent non-muscle invasive bladder cancer. Although PDT showed encouraging therapeutic results, it was largely abandoned due to toxicity or bystander effects on normal cells. The use of a monoclonal antibody-photosensitizer conjugate improves tumor specificity and is a more selective method of delivering light therapy [4].

Enhanced cystoscopy includes technology used to improve the detection of cancers and can reduce the risk of recurrence. Fluorescence has already been used to perform intraoperative analysis of the bladder wall, which was demonstrated in ten patients with superficial bladder cancer. In photodynamic diagnosis (PDD), fluorescence contrast is measured with a video system allowing quantitative assessment of the 5-aminolevulinic acid (5-ALA) accumulation in tissues. The general condition of the bladder can be assessed, the main foci of the tumor can be determined, and a tumor resection can be performed. The procedure is called transurethral resection (TUR). Further examination and identification of the fluorescing boundaries of the tumor remaining after TUR are performed to make a decision of a re-resection. In addition, small fluorescing tumor foci, not detected with white light cystoscopy, can be detected [5].

Raman spectroscopic-based techniques have also been suggested for this application. In contrast to fluorescence, Raman spectroscopy provides a label-free and nondestructive contrast in tissue sections, ex vivo tissue biopsies and in vivo during cystoscopy. The ability to reduce false detection rates in comparison to existing diagnostic tools such as PDD makes Raman spectroscopy particularly attractive as a complementary diagnostic tool. An early in vitro study used a fiber-optic Raman system to collect 220 spectra from 29 fresh frozen bladder samples. A classification algorithm was able to differentiate benign samples from malignant samples with an overall accuracy of 84% [6]. The feasibility of Raman spectroscopy for the in vivo diagnosis was demonstrated using a high-volume-based fiber probe during the TUR of bladder tumors. Multivariate analysis was able to distinguish bladder cancer from normal bladder locations with a sensitivity of 85% and a specificity of 79% [7]. A confocal Raman probe for bladder cancer diagnosis was developed to boost the specificity of the diagnostic algorithm based on its suppression of the out-of-focus non-analyte-specific signals emanating from the neighboring normal tissue. This approach resulted in comparable sensitivity, but significantly higher specificity in relation to high-volume Raman spectral data [8]. A low-resolution fiber-optic Raman spectroscopy system was applied to collect 262 spectra taken from 32 bladder specimens. A principal component-fed artificial neural network categorized the spectra into three groups, namely normal bladder tissue, low-grade and high-grade bladder tumor, with an overall prediction accuracy of 93.1% [9]. An ex vivo study was performed with a combined piezoelectric tube-based optical coherence tomography-probe and fiber-optic probe imaging system that allows large field-of-view imaging of bladder biopsies. Both modalities and co-registered visualization were applied to examine 119 biopsies, and detect and grade cancerous bladder lesions. Raman spectroscopy provided a slightly better sensitivity, but lower specificity for the grading of low- and high-grade tissues than optical coherence tomography [10]. A superficial Raman probe was compared to a non-superficial Raman probe for the in vivo Raman spectroscopy of bladder cancer [11]. Overall, 216 Raman measurements and biopsies were taken in vivo from at least one suspicious and one unsuspicious bladder location in 104 patients. The diagnostic ability was found to be superior using the superficial probe because information is gained solely from the disease’s tissue and irrelevant information from deeper layers is omitted. A combination of label-free quantitative phase imaging of the entire unstained bladder tissue slices and localized Raman spectroscopy was performed. Sparse multinomial logistic regression of the Raman spectra classified the urothelium into benign and malignant bladder cancer tissues with 94.7% [12].

The current study presents a workflow to collect Raman images from control and cancer tissues of ten human bladder resectates and to process the data by unsupervised hyperspectral algorithms vertex component analysis and hierarchical cluster analysis. Due to the high-quality spectra with more than 40 well-resolved bands, various tissue types and further non-tissue materials were identified. This approach offers prospects for clinical translation for intraoperative tissue assessment as a standalone tool or in combination with other modalities to increase the speed and throughput.

## 2. Materials and Methods

### 2.1. Sample Preparation

**Figure 1:**
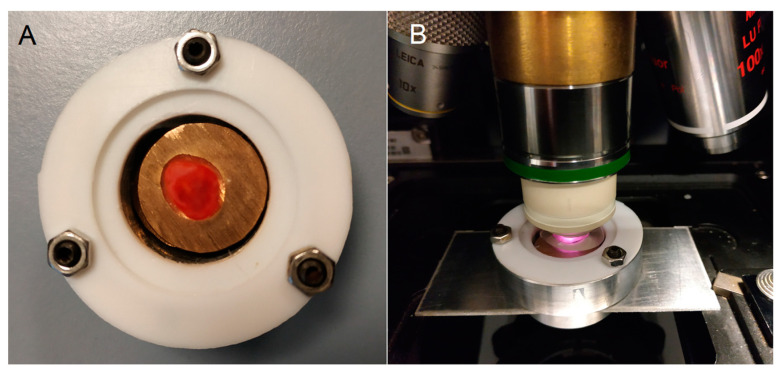
Sample chamber made from a cylindrical aluminum block and covered with a calcium fluoride window. The chamber was sealed by Teflon gaskets and three screws ((**A**), top view). The chamber was mounted with an adapter in a microscope ((**B**), side view). A water droplet was pipetted between the window and water immersion objective lens.

Control and cancer specimens were prepared from ten human bladder resectates from the tissue bank (Comprehensive Cancer Center Freiburg) in Freiburg, shipped on dry ice to Jena and stored at −80 °C until use. Their lateral dimensions ranged from ca. 5–15 mm with a thickness of ca. 5 mm. Samples were placed on copper or silver plates in a sample chamber in the frozen state, thawed at ambient temperature, and covered with a calcium fluoride window to protect them from drying (Figure 1). The height of the plates was adjusted by spacers to achieve gentle contact with the cover window. The sample surface, from which parallel cyrosections were prepared on calcium fluoride windows for Raman and infrared microspectroscopic imaging, faced the top. Results from tissue sections will be presented in subsequent publications. Photographs of all samples were recorded with a digital camera.

### 2.2. Raman Spectroscopy

The Raman spectrometer RXN1 microprobe with a 785 nm laser (Kaiser Optical Systems, Ann Arbor, MI, USA) was used for the acquisition of confocal microscopic data. A water immersion (WI) objective lens 20×/NA 0.5 (Zeiss, Jena, Germany) focused ca. 130 mW intensity onto the sample. Raman images were registered with an exposure time of 5 s per spectrum in a sequential acquisition mode at 250 µm step size using a motorized stage. The instrument was controlled by the software HoloGRAMS, which automatically performs laser wavelength calibration, wavenumber calibration and intensity calibration (Kaiser Optical Systems, Timonium, MD, USA).

### 2.3. Data Processing

Raman images were imported to Matlab (Mathworks, Natick, MA, USA) using the toolbox HoloMap (Kaiser Optical Systems, USA). Without further preprocessing, Raman data were decomposed in the spectral range 600–1800 cm^−1^ by the hyperspectral unmixing algorithm called vertex component analysis (VCA). Briefly, VCA calculates endmember loadings and scores that represent the spectra and concentrations of the most dissimilar components. The number of endmembers was ranged from 4 to 7. The maximum number was achieved when no further component with significant novel features was detected. If components with cosmic spikes occurred, the affected wavenumbers were corrected and the VCA algorithm was executed once more. Details of the implementation of in-house scripts have been described elsewhere [13]. Loadings represented spectral signatures of tissue components that were compiled in a matrix and segmented by hierarchical cluster analysis using the Matlab function “pdist” to calculate the distance matrix, “linkage” to determine the linkage information, and “dendrogram” to display the dendrogram (The Mathworks, Natick, MA, USA). Loadings representing the spectral signature of unknown components were subjected to a Know-it-all (Wiley, Hoboken, NJ, USA) library search.

## 3. Results

### 3.1. Photomicrographs of Bladder Specimens

**Figure 2:**
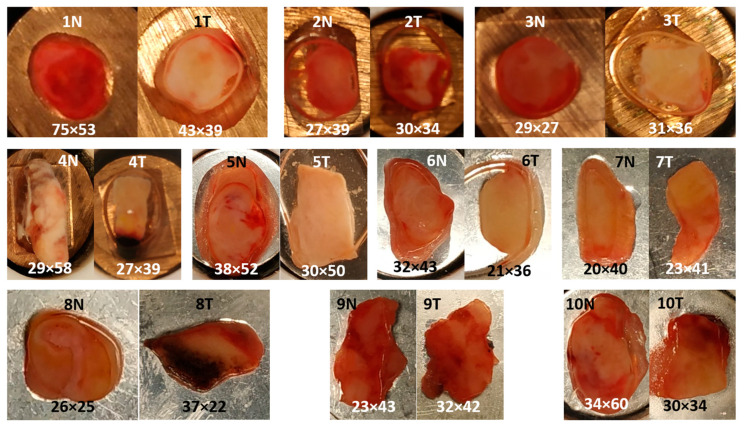
Photomicrographs of control (N) and tumor (T) from 10 resected human bladder. Specimens 1–4 were placed on copper disks, specimens 5–10 on silver disks. Grid size of each Raman map at 250 µm step size is indicated.

Figure 2 shows the photomicrographs of control and tumor specimens from 10 resected human bladders. The liquid around the samples (white in frozen state) is an optimal cutting temperature (OCT) medium in which the specimens were immersed before storage in the tissue bank. Control tissues 1, 2, 3, 5 and 6 appear red, whereas the corresponding cancer tissues 1, 2, 3, 5 and 6 have a brighter hue. Of particular interest is cancer tissue 2 because the bright region in the center is surrounded by a red region. Specimens 7, 9 and 10 have similar appearances for the control and tumor. Tumor tissue 8 shows a dark region from which no usable Raman spectra were collected. Morphological details are evident in control tissue 8. Surprisingly, almost no usable Raman spectra could be obtained here as well. Another dark region was evident in tumor tissue 4, probably due to cauterization. After exclusion of low-quality spectra from this region, the remaining spectra showed normal signal-to-noise ratios and backgrounds. A square region of interest was defined for Raman mapping at a step size of 250 µm. The grid size of each map is indicated.

### 3.2. VCA of Cancer Sample 2

**Figure 3:**
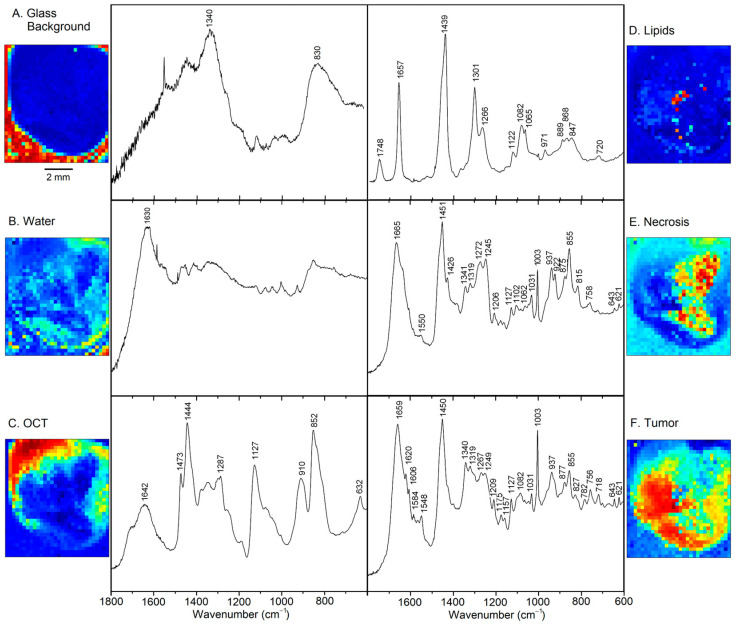
Abundance plots and endmember loadings found in tumor sample 2T by VCA in the spectral range 600–1800 cm^−1^. Abundance plots are scaled from 0 (blue) to 1 (red).

Figure 3 displays the six VCA abundance plots and endmember loadings of sample 2T. The location around the sample and bands near 830 and 1340 cm^−1^ in Figure 3A point to the background of glass components in the beam path, e.g., due to the objective lens. Furthermore, an uncorrected small cosmic spike near 1600 cm^−1^ and elevated noise level between 1400 and 1800 cm^−1^ are evident, which might be a consequence of the relatively weak signal intensity of this component. The band at 1630 cm^−1^ and the location near the tissue margin is typical for spectral contributions of water in Figure 3B. Bands of the OCT medium are characteristic of the transparent liquid in the top portion of sample 2T in Figure 3C. These components could also be detected in almost all specimens and were considered not relevant for tissue analysis.

Figure 3D shows the typical Raman signature of an unsaturated lipids, more specifically a phospholipid such as phosphatidylcholine (PC) [14]. Band assignments are summarized in Table S1 of the supplementary information. These lipids are located near the center of the tissue specimen. Necrosis is detected in the right part of Figure 3E, which coincides with the bright feature in the photomicrograph (Figure 1). The spectral features are assigned to fibrous proteins, mainly collagen [15], which supports this finding. Finally, the tumor is identified in the left part of Figure 3F surrounding necrosis. Spectral features of proteins dominate. However, significant differences are observed compared to the protein bands in Figure 3E, such as weaker collagen bands, more intense aromatic amino acid bands relative to the CH_2_ band near 1450 cm^−1^, and shifts of secondary structure sensitive amide III and amide I bands. Furthermore, smaller spectral contributions were found for the phospholipid PC at 718 and 1082 cm^−1^, and for DNA at 782 cm^−1^ and the shoulder near 1090 cm^−1^. These tissue components are almost unaffected by broad spectral glass background, water and OCT medium, and the spectra only show small baseline slopes towards lower wavenumbers. This justifies that the Raman spectral maps could be processed without preprocessing. The high signal to noise ratio and spectral quality enable detailed analysis of biochemical variations of tumors, which will be described next.

### 3.3. Overview of VCA Result of All Samples

Table 1 summarizes the VCA results of samples 1 to 10. Up to seven components were identified and their tentative assignments are indicated. Spectra from water, glass and OCT medium were almost identical in all samples and were not considered for further analysis, which is focused on Raman spectra of lipids, control, tumor, necrosis and epithelium. Unexpectedly, Raman spectra of carotene, pigments, microplastic and other contaminants were found in 13 out of 19 datasets, which will be described in Section 3.4. Sample 8 only gave poor spectra with high autofluorescence background, weak tissue signals for the normal specimen and no usable spectra for the tumor specimen. The origins of these tissue properties and the extended dark region were not known. The dark region in sample 4T was excluded from the measured region of interest (Figure 1) from which Raman spectra of normal quality were obtained.

**Figure 4:**
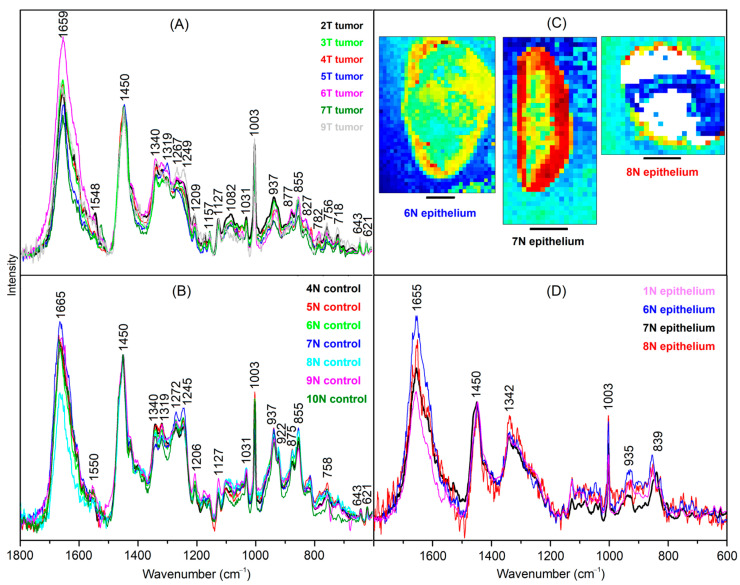
Endmember spectra obtained from VCA of bladder tumor (**A**), control bladder tissue (**B**) and epithelium (**C**). Scale bar = 2 mm (**D**). Spectra are baseline-corrected, normalized and overlaid for comparison. Abundance plots were included for epithelium endmembers.

The tumor spectra of 2T, 3T, 4T, 5T, 6T, 7T and 9T are shown in Figure 4A. The spectra were corrected by subtracting linear baseline segments with minima at 600, 1160, 1500 and 1800 cm^−1^, normalized to equal intensities of the band at 1450 cm^−1^ and overlaid for comparison. The band positions and relative intensities coincide well with the tumor spectrum 2T (plotted in black), which was already shown in Figure 3F. The main variations are observed between 1500 and 1700 cm^−1^, which are assigned to different water content of samples.

The control spectra of 4N, 5N, 6N, 7N, 8N, 9N and 10N are shown in Figure 4B. The spectra were processed in the same way as the tumor spectra. The band positions agree with the necrosis spectrum 2T, which was already shown in Figure 3E. However, bands indicative for fibrous proteins at 815, 855, 922, 937, 1031 and 1245 cm^−1^ are less intense relative to the non-fibrous protein bands, e.g., at 1003 and 1450 cm^−1^. Therefore, this spectrum is typical for the muscle tissue of the bladder wall.

Raman spectra of another tissue type are resolved in 1T, 6N, 7N, and 8N (Figure 4D) that are tentatively assigned to epithelium due to its location near margins (Figure 4C). The spectrum 7N shows the best signal to noise ratio because the margin is broader than for 6N and 8N, where only few weak spectra contribute to the endmember signature. The Raman spectrum of epithelium differs from the spectra of tumor, control and necrosis because amide III bands between 1250 and 1300 cm^−1^ are weak, and bands at 935 cm^−1^ and amide I band at 1655 cm^−1^ point to proteins with α helical secondary structures. Furthermore, a new band arises at 839 cm^−1^.

Lipid spectra of 1N, 2T, 3N, 4N, 4T, 5N, 5T, 6N, 9N, and 9T are shown in Supplementary Figure S1A. The spectra coincide well with the lipid spectrum, which was already shown in Figure 3D. Variations are evident near 1156 and 1525 cm^−1^, which point to carotenoids. More intense carotenoid contributions in specimen 10T are presented in Section 3.4 and Figure S2. Necrosis spectra of 1T, 2T, 5T, and 10T are shown in Supplementary Figure S1B. They are very similar to the necrosis spectrum in Figure 3E.

**Figure 5:**
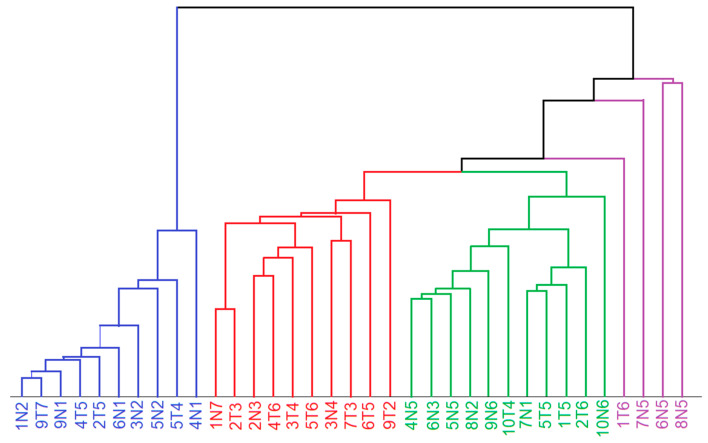
Dendrogram of hierarchical cluster analysis of endmember spectra related to lipids (blue), tumor (red), control and necrosis (green) and epithelium (violet) in the spectral range 800–1380 cm^−1^. Sample nomenclature refer to Table 1.

The lipid- and tissue-related spectra were considered for unsupervised segmentation by hierarchical cluster analysis (HCA). The spectral range from 800 to 1380 cm^−1^ was used as an input for HCA because the weak bands in the range 600–800 cm^−1^ do not significantly contribute to the variations, the band at 1450 cm^−1^ shows only small variations, and the range 1500–1800 cm^−1^ shows unspecific variations due to the water content. The dendrogram in Figure 5 confirms the separation of tumor spectra (Figure 4A) and control tissue spectra (Figure 4B). The Raman spectra of lipid inclusions in samples (Figure S1A) also form a distinct cluster that is well separated from the Raman spectra of tissues with dominant spectral contributions of proteins. Raman spectra from necrosis (Figure S1B) overlap with control spectra, which is consistent with a high abundance of fibrous proteins. The Raman spectra of epithelium (Figure 4D) also form a separate cluster.

### 3.4. Raman Spectra of Microplastics and Pigments in Bladder Tissues

According to Table 1, Raman spectra of carotenes, pigments, microplastic particles and unknown materials were found beside tissue- and non-tissue-related (water, glass, OCT) components in 13 out 19 datasets. As shown in Figure S2, endmembers of low and high carotene content are sparsely distributed in sample 10T, and the characteristic bands at 1525 and 1156 cm^−1^ are clearly evident [16,17]. 

**Figure 6:**
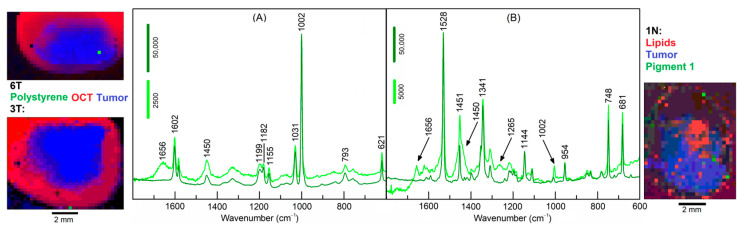
Endmember spectra obtained from VCA of polystyrene in specimens 3T and 6T (**A**) and pigment 1 in specimen 1N (**B**). Color-coded abundance plots show the location of polystyrene and pigment 1 (green), tumor (blue), and optimal cutting temperature medium (OCT) in (**A**) and lipids in (**B**) (red). Whereas pure pigment spectra are obtained at few locations with high intensities (dark green), spectral contributions of tissue are evident in the less intense pigment spectra (light green).

The results of samples 3T and 1N are shown in Figure 6. The loadings of three endmembers were coded with red, green and blue colors to display the distribution of the three selected components. In sample 3T, one pixel at the lower margin next to OCT is assigned to the well-known Raman signature of polystyrene (PS) [18]. The Raman intensities of PS bands are almost 1000 times more intense than the Raman bands of tissue-related materials. The spectral library Know-it-all confirms this assignment with an almost perfect hit quality index (HQI) of 99.7 (Supplementary Figure S3). The Supplementary Figures S3–S10 were reproduced from the Know-it-all reports. In sample 6T, one pixel inside the tumor is also assigned to PS, but at 16-times lower intensities than the PS particle in 3T. Together with the elevated spectral contributions of tissue near 1656 and 1450 cm^−1^, the lower intensities suggest that the PS particle is located below the surface. In sample 1N, several pixels throughout the specimen are assigned to Raman spectra of a pigment with higher and lower intensity (dark and light green, respectively). Here, spectral contributions of tissue are also evident in the lower intense pigment spectrum. The search result identified this pigment as Hostaperm Blue with a hit quality index (HQI) of 58.8 (Supplementary Figure S4). This blue pigment belongs to the chemical class of copper phthalocyanine. The pigment 2 in sample 4N matches with HQI = 71.1 with Terre Verte, which is a green pigment (Supplementary Figure S5). The pigment 3 in sample 6T matches with HQI = 61.8 with Pigment Red 170 (Supplementary Figure S6). The Raman spectra of these pigments are strongly enhanced by the resonance Raman effect, which means the excitation wavelength 785 nm is partly absorbed by the chromophore and the Raman scattering cross section increases if the energy level of the electronic transition agrees with the virtual level of the Raman active-vibrations of the chromophore [17]. However, pigments without resonance enhancement were also detected, such as carbon black [19] in sample 4T with HQI = 91 (Supplementary Figure S7). Residual spectral contributions of the amide I band near 1660 cm^−1^ and CH_2_ band near 1450 cm^−1^ overlap with carbon bands. Another polymer was found in sample 1T with a HQI match of 71.2 to poly (phenylene sulfide) (Supplementary Figure S8). The band positions of the measured spectrum and the library spectrum agreed very well. Deviations occurred for relative band intensities that might be corrected by an intensity calibration procedure, which has recently been described [18]. Although the unknown spectrum in sample 2N gives a reasonable HQI = 68.7 with library entry cellulose acetate sorbate (Supplementary Figure S9), numerous bands deviate, making the assignment less reliable. A cellulose-related fiber from textile material whose spectrum is probably missing in the library is a more likely assignment. Finally, a typical lipid spectrum as presented in Figure 3D was subjected to a library search and yielded an HQI = 85.4 with olive oil (Supplementary Figure S10), which is consistent with similar fatty acid compositions of saturated and unsaturated moieties.

## 4. Discussion

**Figure 7:**
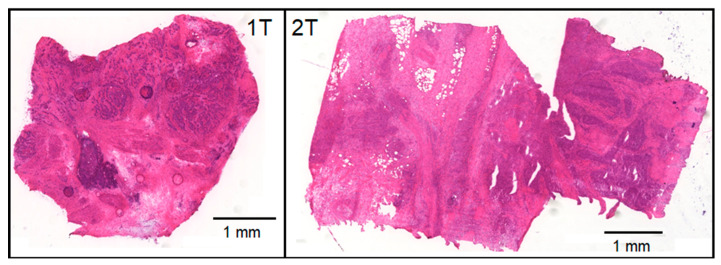
Hematoxylin and eosin stained tissue sections of samples 1T and 2T were cut adjacent to the surface of the specimens that were studied by Raman spectroscopic imaging.

The study demonstrates that Raman spectroscopy provides a wealth of information for tissue assessment and Raman images resolve further spectral and lateral details. Consequently, Raman-based methods were reviewed among other optical and spectral technologies for the clinical need of an intraoperative histopathology [20]. Here, Raman data were collected from bladder specimens and processed by VCA and HCA. The experimental procedure mimics an intraoperative ex vivo scenario where resected tissues are placed in a chamber under a microscope and a coarse map at 250 µm step size and 5 s per spectrum is registered. To speed-up the total acquisition time from more than one hour to a few minutes, exposure time can be reduced, and the step size increased. A water immersion (WI) objective lens with 20× magnification and 0.5 NA gave more intense and better signal-to-noise spectra with a medium penetration depth than 10×/0.25 long working distance air objective lens or 60×/1.0 WI objective lenses that correspond to a high-volume, deeper penetration probe or a low-volume, superficial approach, respectively. A window gave an even sample surface, prevented drying, and a water droplet between the objective lens and window reduced light scattering losses. The mapping resolution and collection depth were sufficient to identify Raman spectra of up to seven components per sample by unsupervised VCA, localize small particles and pigments, and separate non-tissues from tissue components. The tissue components were segmented by HCA and separated lipid inclusion, tumor, necrosis, control tissue and epithelium. Collagenous proteins were the main distinctive feature with low content in tumors, medium content in bladder wall tissue and high content in necrosis. The tumor tissue of specimens 1T, 2T, 5T, 9T and 10T overlapped with the cluster of control tissue, presumably because mixtures of tumors with low content and necrosis with high content of collagenous proteins give spectra that are similar to control bladder wall tissue. This effect is also known as spectral dilution. Raman images would be needed with cellular resolution to improve the separation of tumor and necrosis, to visualize cell nuclei and increased cell nuclei density in tumors. Figure 7 shows parallel hematoxylin and eosin (H&E) stained tissue sections of samples 1T and 2T that confirm larger and smaller tumor areas that are surrounded by necrosis. Empty holes in the left part of 2T are consistent with lipid deposits that were detected in the Raman image of Figure 2 and were washed away in H&E stained tissue sections by solvents during the staining procedure.

Higher resolution Raman images at 2 µm step size from smaller regions of interest were previously reported for primary brain tumor sections immersed in aqueous buffer [21] that demonstrated the separation of tumor and necrosis based on chemical and morphological features. For quantitation of brain tumor composition, VCA was performed on the pooled Raman images, representative endmember spectra for proteins, nucleic acids, cholesterol, and phospholipids were derived and used for non-negative least square fitting of Raman spectra. This approach is expected to also give more robust results for bladder cancer compared to analysis based on single Raman images. A prerequisite is Raman images of more specimens that will be collected in future studies.

The observation that lipid inclusions were found both in normal and tumor specimens suggests that their presence or absence cannot be considered as tumor markers. Besides the depletion of collagenous proteins, spectral contributions of DNA and the phospholipid phosphatidylcholine were identified as tumor markers. Whereas DNA is not found in non-cancer bladders, increased DNA content is associated with a high density and proliferation rate of cancer cells, which are among the hallmarks of cancer [22]. A review described altered phosphatidylcholine synthesis in cancer, and how these changes contribute to malignant growth [23]. Although specimens were selected from tumor and tumor-free regions, control spectra 1N7, 2N3 and 3N4 were assigned to cancer according to the HCA dendrogram. Careful inspection of 2N3 (Figure S11) revealed the presence of DNA and phosphatidylcholine Raman bands as tumor markers. Distant tumor locations might be consistent with malignant, high grade tumors. Similarly, the spectra 1N7 and 3N4 (Figure S11) were clearly distinct from normal bladder tissue and show a typical tumor signature except for less intense DNA bands near 784 and 1086 cm^−1^, which might point to pre-malignant tissue or another non-normal tissue type.

The assessment of water content in fresh specimens by Raman spectroscopy was proposed as an intraoperative tool. In particular, changes in water content were quantified by measuring the water of the total area of the high wavenumber range between 2800 and 3600 cm^−1^ and differentiated between tumor and non-tumor breast tissue [24] and oral tissue [25]. Variations in water content were not considered relevant in this study because the specimens were not fresh and variations of water content during storage, transport and preparation could not be excluded.

An unexpected result was the detection of carotenes, microplastic particles and pigments in bladder samples. The VCA algorithm has sufficient sensitivity and specificity to identify even single pixels with Raman spectra of foreign material. The high spectral quality and signal to noise ratio allowed the direct import of the unknown spectra to a library and gave, in most cases, reasonable assignments. Carotenes has previously been detected by Raman spectroscopy in thin brain tumor sections immersed in buffer using similar unsupervised hyperspectral unmixing approaches [16]. The ability of Raman spectroscopy to identify microplastic particles from environmental samples such as water, soil and food is well known [26]. Although nano- and microplastic pollution has emerged as a global issue, uptake in the animal models and humans is controversial and only a few studies have been reported so far. Male mice received a single administration of fluorescent polystyrene beads of 100 nm and 3 µm diameter via tail vein injection, gavage, or pulmonary perfusion, and both particles were detected in urine by confocal laser scanning microscopy and transmission electron microscopy [27]. Microplastic has been detected in human placenta and meconium in a clinical setting [28]. The detection of microplastic particles, pigments and other foreign materials in bladder cancer specimens has not been reported yet and several explanations are feasible.

First, plastic pollution is taken up via food, drinks or inhalation of air, and accumulates in the bladder while only a fraction is excreted with the urine. The frequent detection of such foreign particles in this work (13 out of 19 specimens from resected bladders!) might point to a factor in the development of malignancies and might have been overlooked so far by state-of-the art modalities such as histopathology, immunohistochemistry and fluorescence that usually administer stains to enhance contrast. Advantages of Raman spectroscopy include that (i) no stains are required, (ii) unprocessed tissue can be analyzed, (iii) algorithms can be applied without complex data pre-processing such as VCA in this and earlier work [13,16,21], and (iv) even in vivo applications are possible using fiber optic probes [6,7,8,10,11,29].

Second, pigments such as phthalocyanine derivatives are used as photosensitizers in PDT, which is also a treatment option in bladder cancer. Photosensitizers are designed so that they accumulate in cancer after administration and degrade upon local illumination using a cystoscope to a new chemical that kills cancer cells. Non-degraded photosensitizers might explain the more frequent occurrence of pigments in non-illuminated control specimens 1N, 4N, 5N, 7N, 8N and 9N than in cancer specimens 6T and 7T.

Third, blue, green, red and black pigments are popular tattoo inks, which are injected into the top layer of the skin dermis (ca. 1–2 mm) by needles, and might enter the blood stream and accumulate in organs as already observed in the liver [30]. The potential role of tattoo inks in bladder cancer was also reported [31].

Finally, contaminations during the course of tissue collection at the operating theater and transfer to the Raman spectroscopy lab might occur, e.g., placing samples in Petri dishes made of polystyrene and cellulose fibers originating from papers. Therefore, preparation and isolation protocols that have been developed for microplastics under carefully controlled (ideally clean-room) conditions from food matrices [32] need to be adapted to future bladder tissue studies. Owing to the penetration limit of the Raman spectroscopic configuration in the range of hundreds of micrometers, deeper plastic and pigment materials could not be detected and their total amount might be underestimated.

## 5. Conclusions

Our Raman workflow and data processing protocol with minimal user interference and sample preparation offers advantages for future clinical translation such as intraoperative tumor detection and label-free material identification in complex matrices. Here, experimental data from a relatively small cohort of 10 control and 10 tumor samples of resected bladders already revealed a plethora of information encompassing more than 40 Raman bands in the fingerprint range 600–1800 cm^−1^, five tissue components (control, tumor necrosis, lipids and epithelium), three non-tissue components (water, glass and OCT), and more than eight additional components (carotene, blue/green/red/black pigments, PS and PPS microplastics plus more yet unknown materials). Further studies about the origin, the identity and the quantity of foreign materials in bladder tissues are needed to check the hypotheses with more patients or animal models, anonymous metadata (treatment history, tattoos, etc.) and careful preparation protocols to minimize possible contaminations. Due to its label-free nature and high specificity, Raman spectroscopic imaging is well suited for such studies, ideally in multiple centers under standardized conditions, and gives unique insights that can complement the established clinical modalities.

## Figures and Tables

**Table 1:** Tentative assignment of tissue components obtained by VCA-based unmixing of Raman images from normal (N) and tumor (T) specimens 1–10.

	VC1	VC2	VC3	VC4	VC5	VC6	VC7
1N	Pigment 1	Lipid	Water	OCT	Pigment 1	Glass	Tumor
1T	PPS	Lipid/protein	Glass	OCT	Necrosis	Epithelium	Water
2N	Unknown	OCT	Tumor	Water	Glass		
2T	Glass	Water	Tumor	OCT	Lipid	Necrosis	
3N	Glass	Lipid	Water	Tumor			
3T	PS	Glass	OCT	Tumor			
4N	Lipid	Pigment 2	Water	Glass	Control	OCT	
4T	Glass	Unknown	OCT	Carbon	Lipid	Tumor	
5N	Pigment 1	Lipid	Glass	Water	Control		
5T	Glass	Water	OCT	Lipid	Necrosis	Tumor	
6N	Lipid	Water	Control	Glass	Epithelium		
6T	Pigment 3	PS	Glass	OCT	Tumor	Water	
7N	Control	Glass	Water	Pigment	Epithelium		
7T	Pigment 1	Glass	Tumor	Pigment 1	Tumor		
8N	Pigment 1	Control	Glass	Water	Epithelium		
8T							
9N	Lipid	Glass	Noise	Water	Pigment 1	Control	
9T	Noise	Tumor	Noise	Noise	Glass	Water	Lipid
10N	Lipid/protein	Glass/OCT	OCT	Water	Glass	Control	
10T	Glass	Lipid/protein	unknown	Necrosis	Carotene	Water	Carotene

PPS: Poly (phenylene sulfide), PS: Polystyrene, OCT: optimal cutting temperature medium. Shading indicates microplastic, pigments and contaminants.

## Data Availability

Raw data will be provided upon request.

## Supplementary Materials

The following supporting information can be downloaded at: https://www.mdpi.com/article/10.3390/cancers15072162/s1, Figure S1: Endmember spectra of lipid (A) and necrosis (B). Nomenclature refer to Table 1 in the main text. Table S1: Positions and assignments of labeled Raman bands in endmember spectra of lipids, tumor and necrosis in Figure 2 of the main text. The bands in necrosis are also found in control bladder at lower intensities in Figure 3. Figure S2: Endmember spectra of sample 10T with low and high spectral contributions of carotenoids. Figure S3: HQI = 99.7 between polymer spectrum in sample 3T (black) and polystyrene (black) from spectral library. Figure S4: HQI = 58.8 between pigment 1 spectrum in sample 1N (black) and Hostaperm blue (red) from spectral library. Figure S5: HQI = 71.1 between pigment 2 spectrum in sample 4n (black) and Terre Verde (red) from spectral library. Figure S6: HQI = 61.8 between pigment 3 spectrum in sample 6T (black) and pigment red (red) from spectral library. Figure S7: HQI = 91 between carbon spectrum in sample 4T (black) and carbon black (red) from spectral library. Figure S8: HQI = 71.2 between polymer spectrum in sample 1T (black) and poly(phenylene sulfide) (red) from spectral library. Figure S9: HQI = 68.7 between unknown spectrum in sample 2N (black) and cellulose acetate sorbate (red) from spectral library. Figure S10: HQI = 85.4 between lipid spectrum in sample 3N (black) and olive oil (red) from spectral library. Figure S11: Raman spectra of normal specimens that were clustered with tumor spectra: 1N7 (blue), 2N3 (red) and 3N4 (green) [14,17,33,34].
