# Supplementary material for: Raman Spectroscopic Imaging of Human Bladder Resectates towards Intraoperative Cancer Assessment
Source: Cancers (Basel). 2023 Apr 5;15(7):2162. doi: 10.3390/cancers15072162 (PMC10093366; doi:10.3390/cancers15072162)
Supplement: Supplementary file 1 [file cancers-15-02162-s001.zip › cancers-2157913-supplementary.pdf]

cancers

MDPI

Supplementary information

Raman Spectroscopic Imaging of Human Bladder Resectates towards Intraoperative Cancer Assessment

Christoph Krafft [1,*], Jürgen Popp [1,2], Peter Bronsert [3] and Arkadiusz Miernik [4]

1 Leibniz Institute of Photonic Technology, Member of Leibniz Health Technologies and Member of the Leibniz Centre for Photonics in Infection Research, 07745 Jena, Germany
2 Institute of Physical Chemistry and Abbe Center of Photonics, Friedrich Schiller University Jena, Member of the Leibniz Centre for Photonics in Infection Research, 07743 Jena, Germany
3 Medical Center, Faculty of Medicine, Institute of Surgical Pathology, University of Freiburg, 79106 Freiburg, Germany
4 Medical Center, Faculty of Medicine, Department of Urology, University of Freiburg, 79106 Freiburg, Germany
* Correspondence: christoph.krafft@leibniz-ipht.de; Tel.: +49-3641-206306

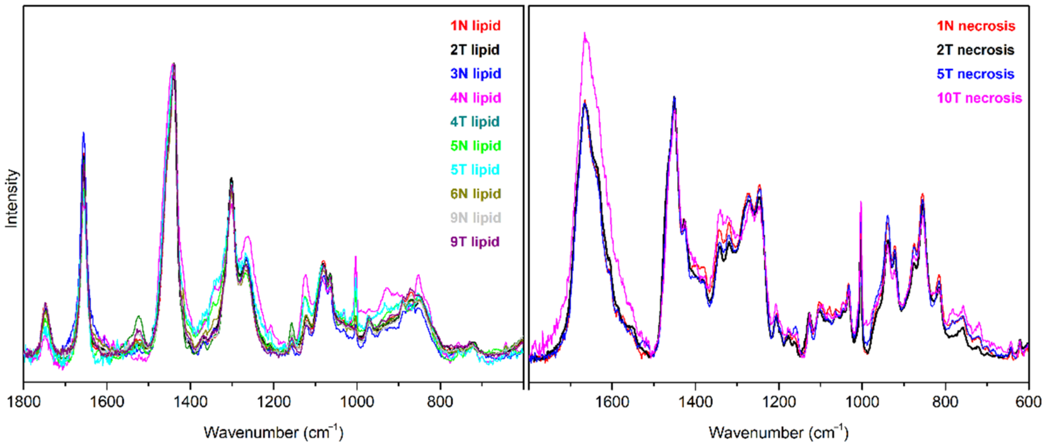

Figure S1: Endmember spectra of lipid (A) and necrosis (B). Nomenclature refer to table 1. Band positions agree with the spectra in Figure 3.

Lipid $[cm^{-1}]$	Assignment [1]	Tumor $[cm^{-1}]$	Assignment [2]	Tumor $[cm^{-1}]$	Assignment [2]	Necrosis $[cm^{-1}]$	Assignment [3]
1748	ν(C=O)	1659	Amide I	1127	ν(C-C)	1665	Amide I
1657	ν(C=C)	1620	Trp/Tyr	1082	$\nu_s(PO_4^{2-})$	1426	δ(CH_2)
1439	δ(CH_2)	1606	Phe	1031	Phe	1272	Amide III
1301	δ(CH_2)	1584	Phe	1003	Phe	1245	Amide III
1266	ν(C=C-H)	1548	Trp	937	Bk(C-C_α)	1031	Hydroxy-pyridinium
1122	ν(C-C)	1450	δ($CH_{2/3}$)	877	Trp		
1082	$\nu_s(PO_4^{2-})$	1340	δ($CH_{2/3}$)	855	Tyr	937	Bk(C-C_α)
1065	ν(C-C)	1319	δ($CH_{2/3}$)	827	Tyr	922	Bk(C-C_α)
971	Backbone	1267	Amide III	782	T, C	875	Hydroxy-proline
889	Backbone	1249	Amide III	756	Trp	855	
868	Backbone	1209	Trp	718	$N(CH_3)_3$	812	Proline
847	Backbone	1175	Tyr	643	Tyr		
720	$N(CH_3)_3$	1157	ν(C-N)	621	Phe		

Table S1: Positions and assignments of labeled Raman bands in endmember spectra of lipids, tumor and necrosis in figure 2 of the main text. The bands in necrosis are also found in control bladder at lower intensities in figure 3. Most protein related bands in tumor are also observed in necrosis, however at lower relative intensities. They were not listed in the last column for brevity

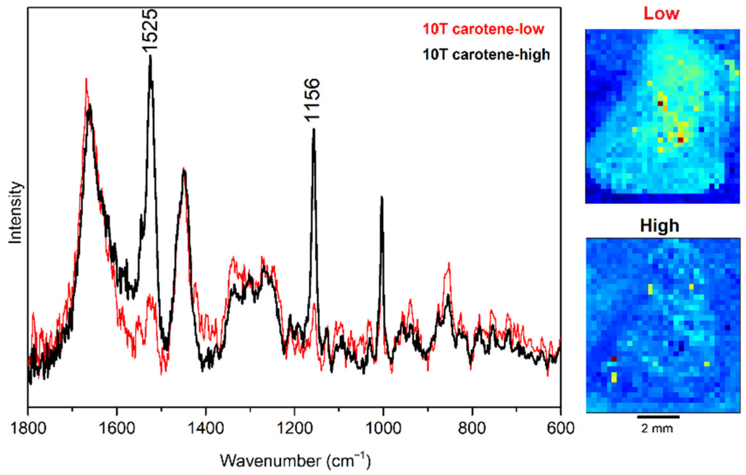

Figure S2: Endmember spectra of sample 10T with low and high spectral contributions of carotenoids. [1,4]

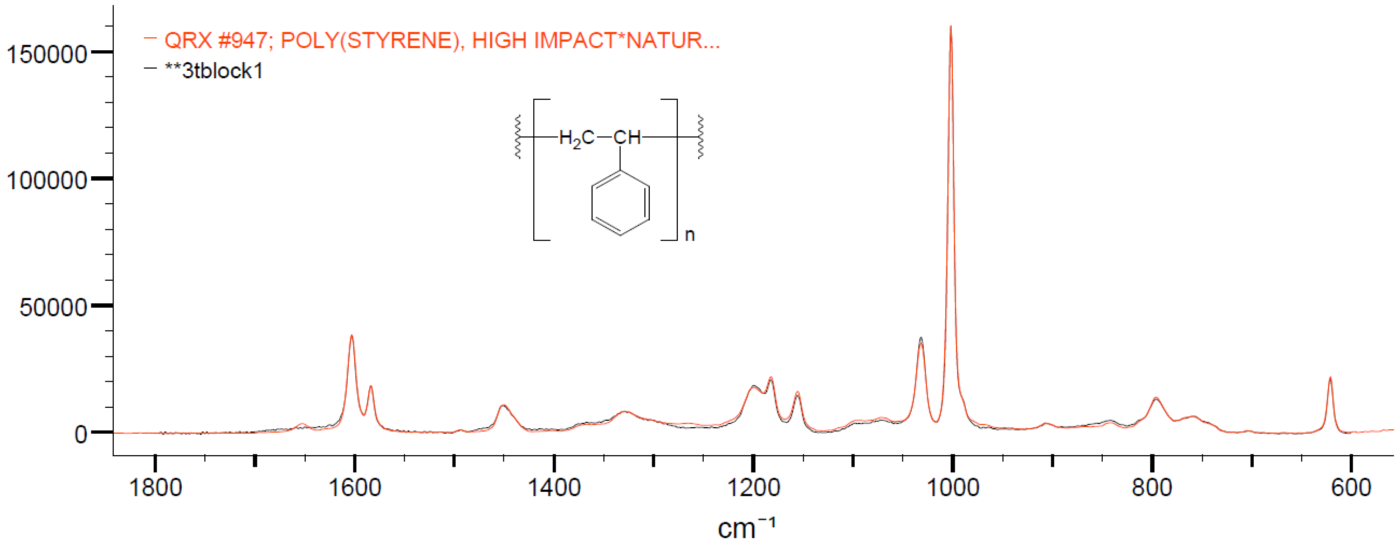

Figure S3: HQI=99.7 between polymer spectrum in sample 3T (black) and polystyrene (black) from spectral library

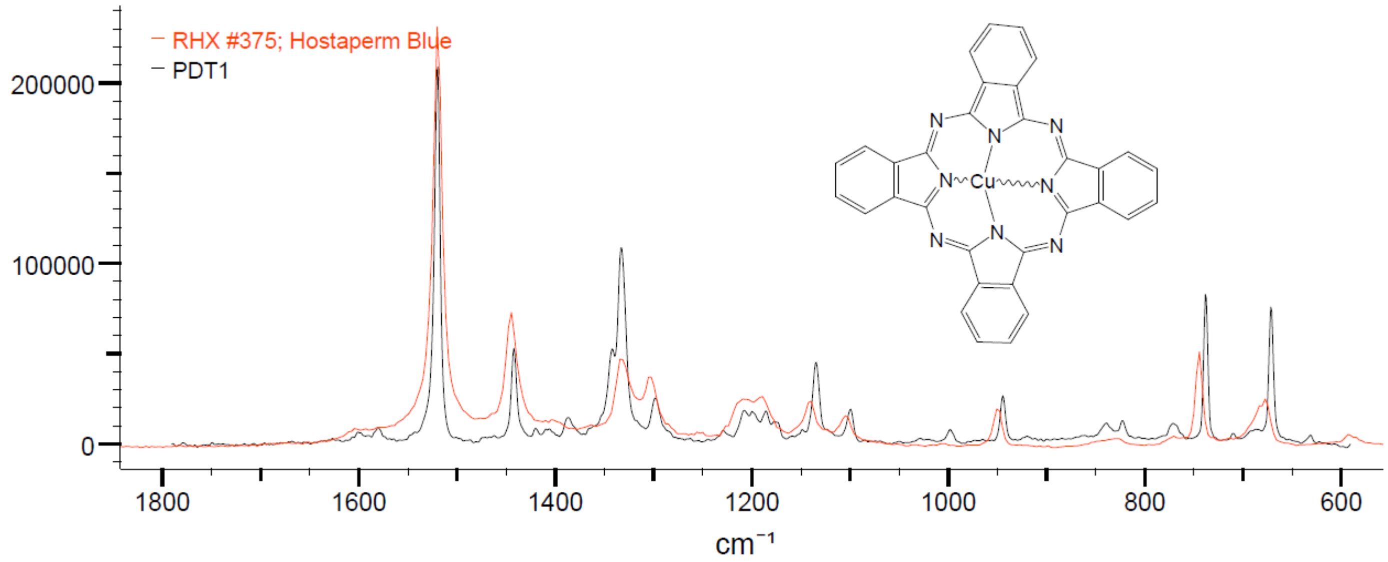

Figure S4: HQI=58.8 between pigment 1 spectrum in sample 1N (black) and hostaperm blue (red) from spectral library

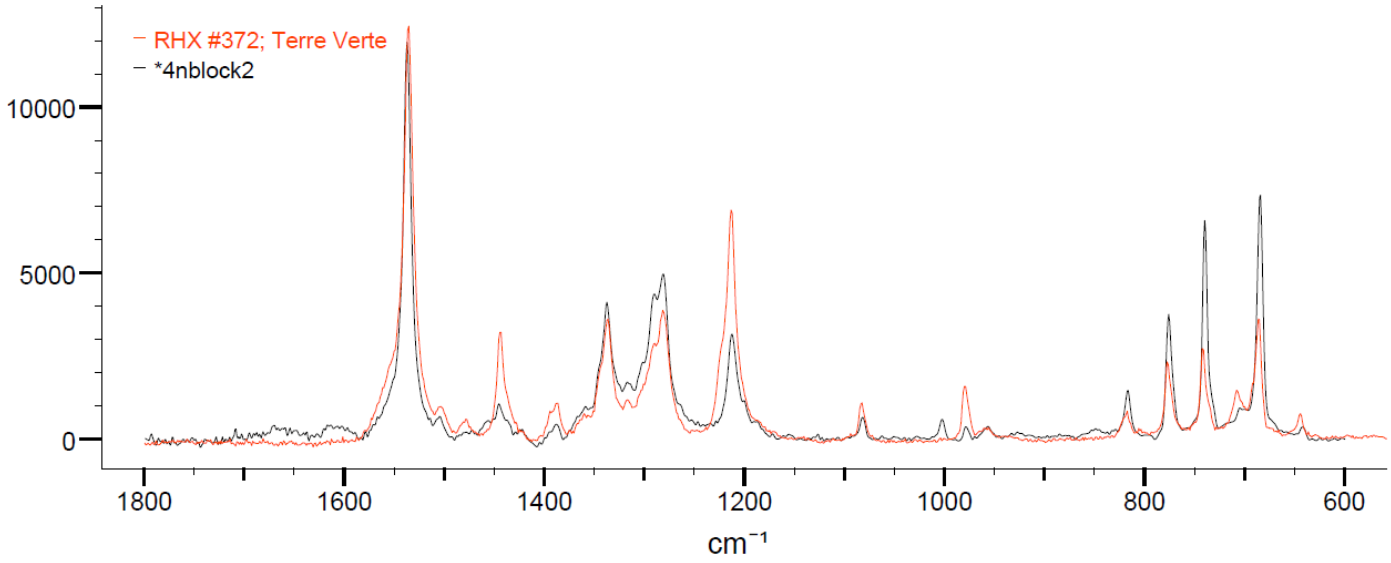

Figure S5: HQI=71.1 between pigment 2 spectrum in sample 4n (black) and terre verde (red) from spectral library

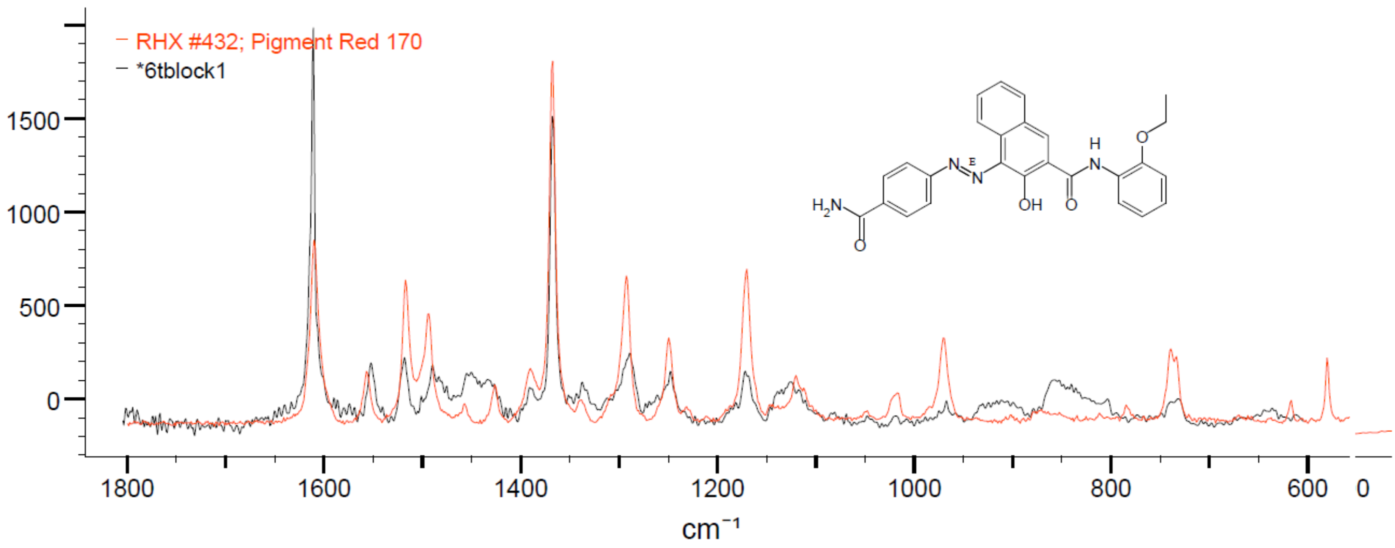

Figure S6: HQI=61.8 between pigment 3 spectrum in sample 6T (black) and pigment red (red) from spectral library

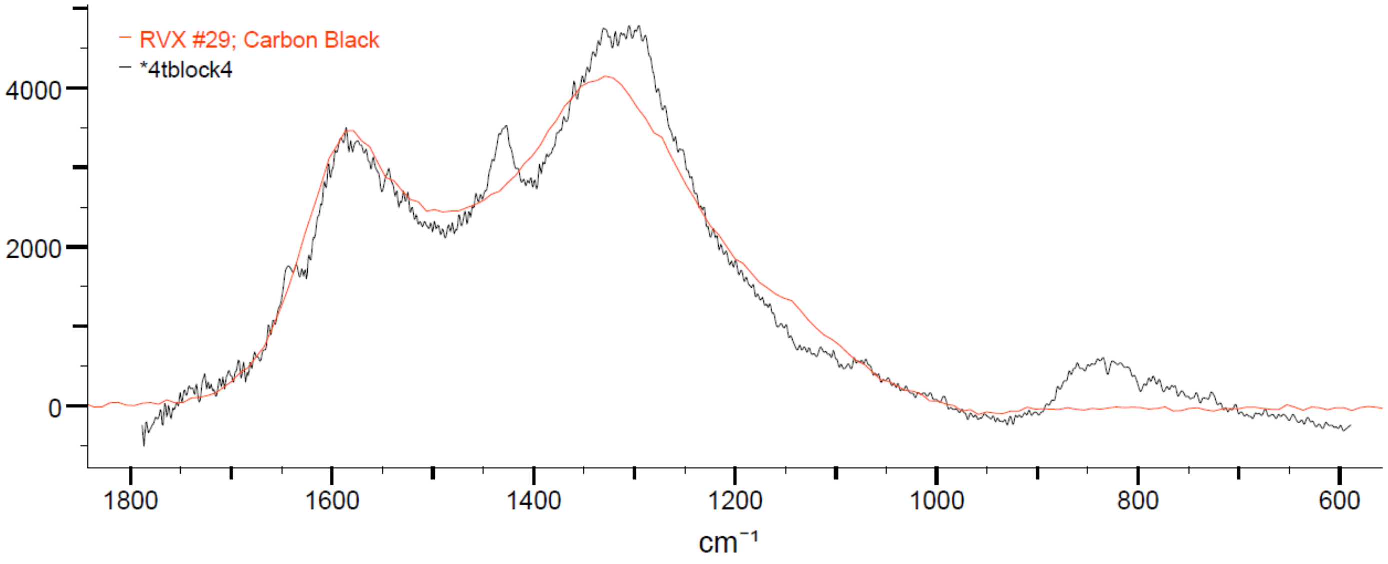

Figure S7: HQI=91 between carbon spectrum in sample 4T (black) and carbon black (red) from spectral library

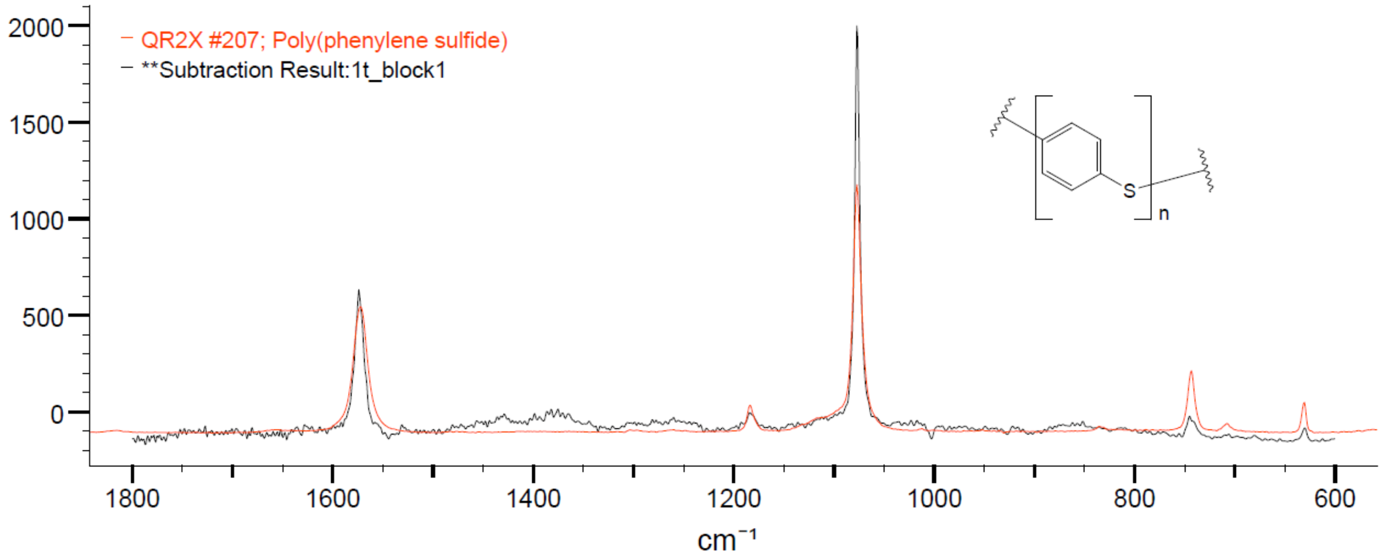

Figure S8: HQI=71.2 between polymer spectrum in sample 1T (black) and poly(phenylene sulfide) (red) from spectral library

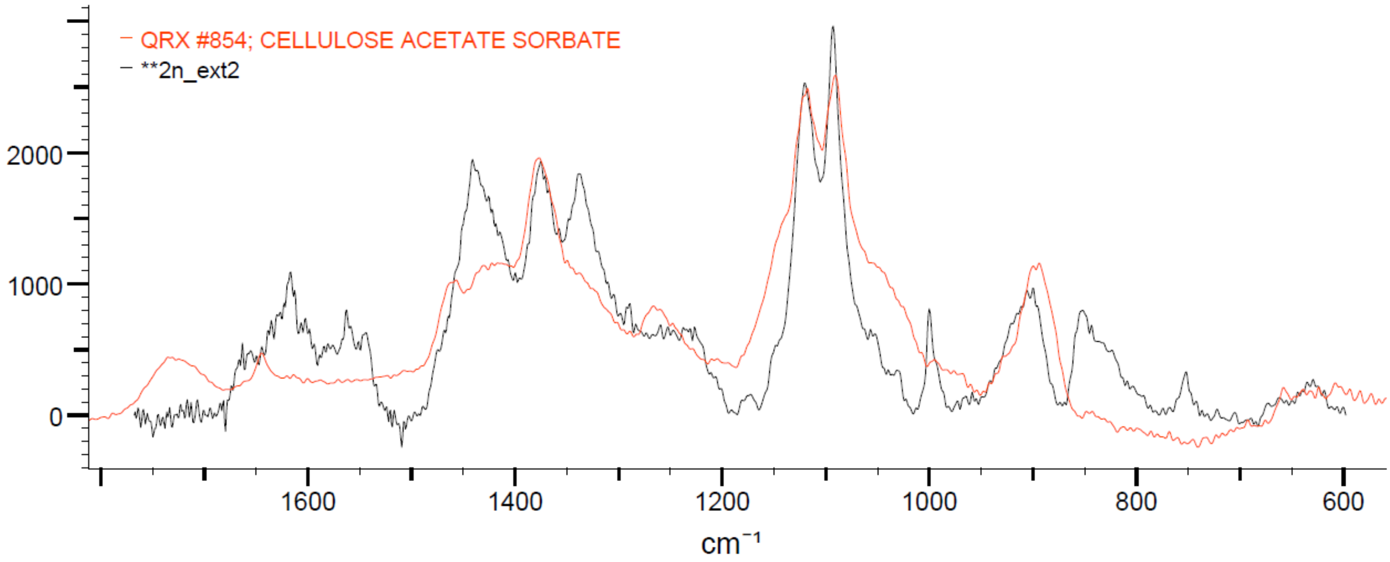

Figure S9: HQI=68.7 between unknown spectrum in sample 2N (black) and cellulose acetate sorbate (red) from spectral library

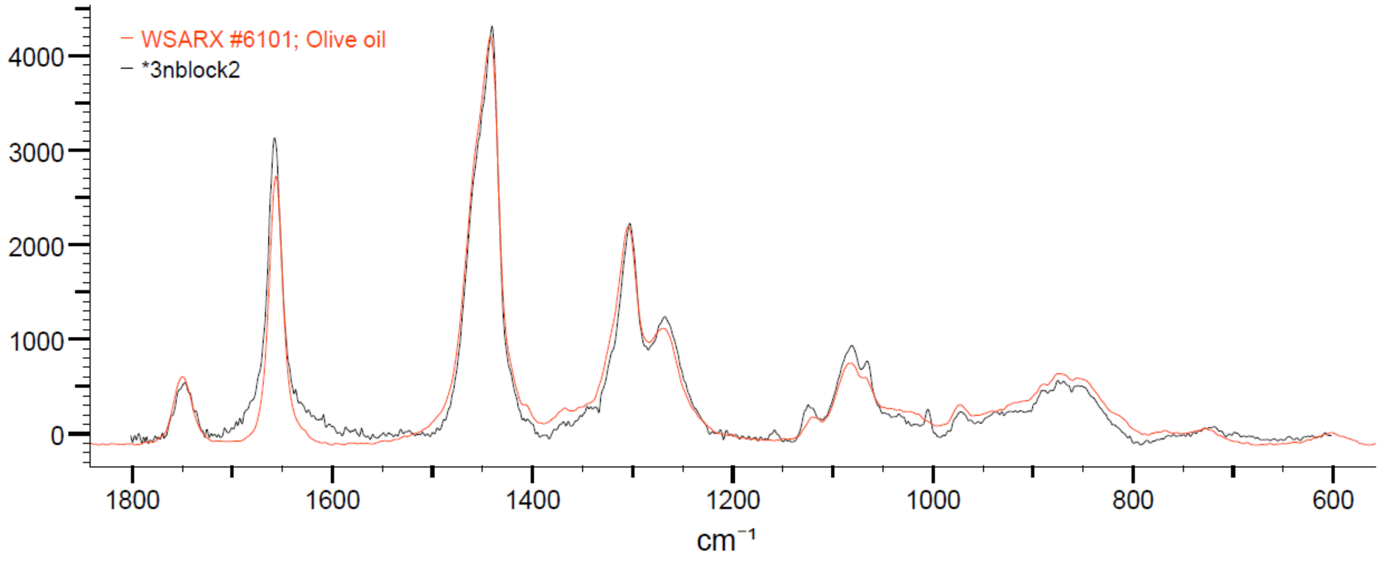

Figure S10: HQI=85.4 between lipid spectrum in sample 3N (black) and olive oil (red) from spectral library.

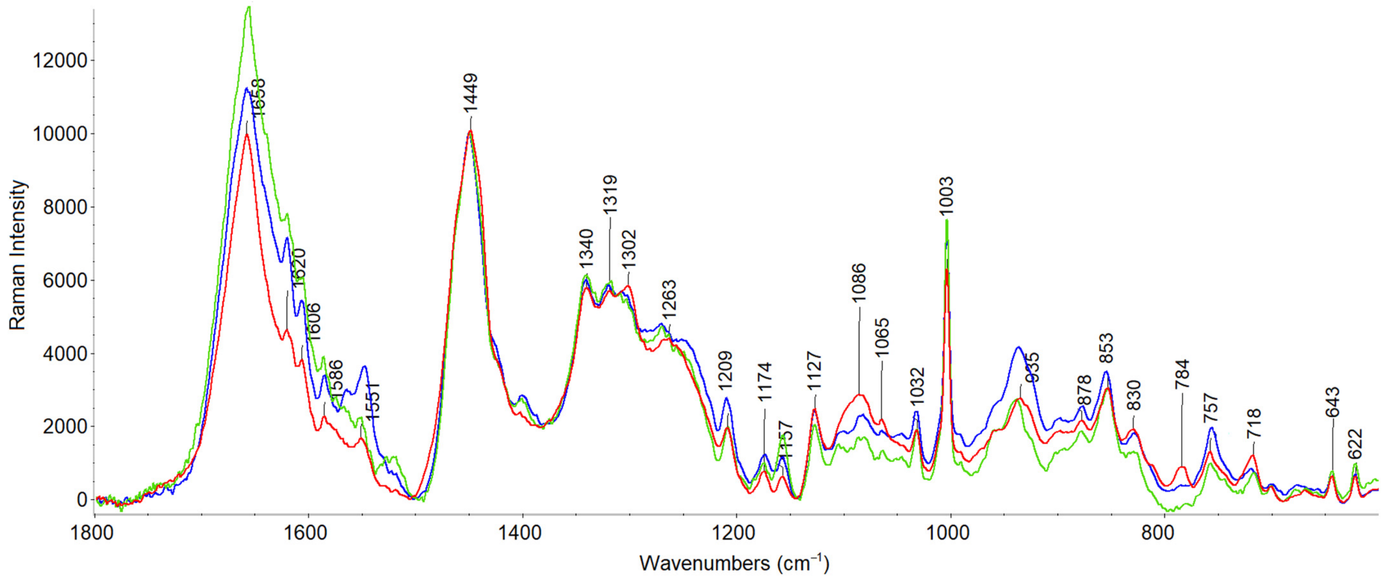

Figure S11: Raman spectra of normal specimens that were clustered with tumor spectra: 1N7 (blue), 2N3 (red) and 3N4 (green).

References:

1. Krafft, C.; Neudert, L.; Simat, T.; Salzer, R. Near infrared Raman spectra of human brain lipids. *Spectrochimica Acta Part A: Molecular and Biomolecular Spectroscopy* **2005**, *61*, 1529-1535, https://doi.org/10.1016/j.saa.2004.11.017
2. Krafft, C. Raman Spectroscopy of Proteins and Nucleic Acids: From Amino Acids and Nucleotides to Large Assemblies. In *Encyclopedia of Analytical Chemistry*, **2018**, pp. 1-15, https://doi.org/10.1002/9780470027318.a9611
3. Shaik, T.A.; Alfonso-García, A.; Zhou, X.; Arnold, K.M.; Haudenschild, A.K.; Krafft, C.; Griffiths, L.G.; Popp, J.; Marcu, L. FLIm-Guided Raman Imaging to Study Cross-Linking and Calcification of Bovine Pericardium. *Analytical Chemistry* **2020**, *92*, 10659-10667, https://doi.org/10.1021/acs.analchem.0c01772
4. Krafft, C.; Schmitt, M.; Schie, I.W.; Cialla-May, D.; Matthäus, C.; Bocklitz, T.; Popp, J. Label-Free Molecular Imaging of Biological Cells and Tissues by Linear and Nonlinear Raman Spectroscopic Approaches. *Angewandte Chemie International Edition* **2017**, *56*, 4392-4430, https://doi.org/10.1002/anie.201607604
